# Silicon Carbide and MRI: Towards Developing a MRI Safe Neural Interface

**DOI:** 10.3390/mi12020126

**Published:** 2021-01-26

**Authors:** Mohammad Beygi, William Dominguez-Viqueira, Chenyin Feng, Gokhan Mumcu, Christopher L. Frewin, Francesco La Via, Stephen E. Saddow

**Affiliations:** 1 Electrical Engineering Department, University of South Florida, Tampa, FL 33620, USA; mbeygi@usf.edu (M.B.); mumcu@usf.edu (G.M.); 2 Moffitt Cancer Center, Tampa, FL 33620, USA; Will.Dominguez.V@gmail.com; 3 Department of Mechanical Engineering, University of South Florida, Tampa, FL 33620, USA; chenyinfeng@usf.edu; 4 NeuroNexus, LLC, Ann Arbor, MI 48108, USA; cfrewin@neuronexus.com; 5 IMM-CNR, Catania, I-95121 Sicily, Italy; francesco.lavia@imm.cnr.it; 6 Department of Medical Engineering, University of South Florida, Tampa, FL 33620, USA

**Keywords:** MRI compatibility, silicon carbide, neural interface, SAR, finite element simulation, MRI image artifacts

## Abstract

An essential method to investigate neuromodulation effects of an invasive neural interface (INI) is magnetic resonance imaging (MRI). Presently, MRI imaging of patients with neural implants is highly restricted in high field MRI (e.g., 3 T and higher) due to patient safety concerns. This results in lower resolution MRI images and, consequently, degrades the efficacy of MRI imaging for diagnostic purposes in these patients. Cubic silicon carbide (3C-SiC) is a biocompatible wide-band-gap semiconductor with a high thermal conductivity and magnetic susceptibility compatible with brain tissue. It also has modifiable electrical conductivity through doping level control. These properties can improve the MRI compliance of 3C-SiC INIs, specifically in high field MRI scanning. In this work, the MRI compliance of epitaxial SiC films grown on various Si wafers, used to implement a monolithic neural implant (*all*-SiC), was studied. Via finite element method (FEM) and Fourier-based simulations, the specific absorption rate (SAR), induced heating, and image artifacts caused by the portion of the implant within a brain tissue phantom located in a 7 T small animal MRI machine were estimated and measured. The specific goal was to compare implant materials; thus, the effect of leads outside the tissue was not considered. The results of the simulations were validated via phantom experiments in the same 7 T MRI system. The simulation and experimental results revealed that free-standing 3C-SiC films had little to no image artifacts compared to silicon and platinum reference materials inside the MRI at 7 T. In addition, FEM simulations predicted an ~30% SAR reduction for 3C-SiC compared to Pt. These initial simulations and experiments indicate an *all*-SiC INI may effectively reduce MRI induced heating and image artifacts in high field MRI. In order to evaluate the MRI safety of a closed-loop, fully functional *all*-SiC INI as per ISO/TS 10974:2018 standard, additional research and development is being conducted and will be reported at a later date.

## 1. Introduction

The utilization of neuromodulation in the treatment of neurological disorders, such as chronic pain, Parkinson’s disease, tremors, and dystonia, has been growing over the past decade [1]. For many of these treatments, magnetic resonance imaging (MRI) is used as a means to map the region of interest for implant placement inside the body and to monitor the post-operation/treatment condition [2]. In addition, MRI is a common noninvasive diagnostic tool that produces high-contrast images of soft tissue while avoiding the use of ionizing radiation. Unfortunately, “MR-conditional” implants are limited to a static magnetic field (**B_0_**) strength of 1.5 T for most cases [3], and recently in a few cases to 3 T [4], to ensure patient safety. Ultra-high field MRI (**B_0_** ≥ 7 T) produces higher resolution images, with higher SNR and CNR, and improves existing MRI applications such as high-resolution structural and susceptibility-weighted imaging [5].

There are certain complications that occur when an implant containing conductive or magnetic material is placed in an MRI machine. Image artifacts make post-implantation monitoring practically impossible, thus limiting the use of conductive and magnetic materials in neural implants [6]. A neural implant can interact with the static magnetic field (**B_0_**), gradient magnetic fields (d**B**/dx), and radio frequency (RF) fields (**B_1_**) in an MRI machine. These interactions can damage the surrounding tissue by inducing mechanical vibrations and heat, as reported in [1,3,7,8,9,10]. They also reduce image quality by introducing image artifacts through implant interaction with **B_0_** and **B_1_** fields and thus complicate image interpretation [6,11,12,13,14].

During the past decades, several studies have focused on the safety of implants under MRI with several techniques proposed to overcome the safety issue [10,14,15,16,17,18,19]. Reported approaches to develop a more MRI compatible neural implant are mainly based on low electrically conductive materials, materials with magnetic susceptibility compatible with tissue, and the shielding of electrodes with high dielectric materials or conductive coils [17,18,20,21,22,23,24,25].

Regarding the heat induced in implanted devices by the gradient magnetic and RF fields, several in-vitro and in-vivo simulation studies have been conducted in order to estimate the temperature rise induced by each MRI scan [23,26,27,28,29,30,31]. The range of the temperature rise reported by each study is widely diverse and essentially dependent on the implant materials studied, their geometry, and the magnetic field frequency and intensity used.

Reported in [32], the severity of the image artifacts caused by a magnetic susceptibility difference (Δχ) in metallic materials and silicon, with tissue, was investigated. This study showed that a materials’ magnetic susceptibility (χ) plays an important role in defining the intensity of the image artifacts under MRI.

In a recent study [14], glassy carbon (GC) microelectrodes were introduced that showed less image artifacts, lower displacement, and lower interaction with RF fields under MRI scans in a tissue phantom. The GC electrode conductivity was low, in the range of ~7 × 10^3^ S/m, in comparison with platinum (Pt), which is ~9 × 10^6^ S/m. In addition, due to the negative magnetic susceptibility of GC (χ = –1.2 ppm), it is believed that the artifacts caused by Δχ were minimized [14].

Although many of the approaches mentioned above showed acceptable MRI compatibility, a robust monolithic INI with high bio- and hemocompatibility that also displays a high level of MRI compatibility, is absent in the literature.

Cubic silicon carbide (3C-SiC) is a wide band gap (E_G_ = 2.3 eV) semiconductor that has been proven to have a high compatibility with neural tissue [33]. The mechanical, chemical, and electrical properties of SiC as a class of materials (there are numerous single-crystal forms, such as 3C-SiC, as well as poly- and amorphous forms), as well as its high biocompatibility, have made SiC an attractive material for fabricating neural implants [34,35,36,37,38,39,40,41]. 3C-SiC possesses a modifiable electrical conductivity through intentional atomic doping during material processing [42] that can reduce the magnetic coupling with time-varying magnetic fields in MRI scans.

SiC also has a magnetic susceptibility, *χ*, of −12.87 ppm, which is close to human tissue (*χ* ~ −9 ppm) compared to platinum (Pt) (*χ* = 267 ppm) and Si (*χ* = −3.24 ppm). However, the actual magnetic susceptibility value in semiconductors depends on their doping type (n or p) and doping densities [43,44].

Magnetic field perturbation may occur during a scan when there is a foreign object present that has a different magnetic susceptibility than that of the tissue (i.e., Δχ). In this case, as the implant has a different χ than tissue, a much larger frequency shift may occur (as large as 100 Hz for each ppm under 7 T [45]). This frequency shift causes artifacts to be displayed on the resulting MRI images [45]. In order to minimize these image artifacts, the implant should be fabricated with a material whose χ is as close as possible to that of the surrounding tissue.

When one considers relatively moderate-resistivity conductive traces, in comparison with metals, neural probes fabricated monolithically from 3C-SiC may reduce image artifacts by reducing the **B_1_** field distortion caused by highly conductive materials such as noble metals. In addition, the lower electron concentration of highly doped n-type 3C-SiC (~10^19^ vs. ~ 10^23^ cm^−3^ for Pt) decreases the level of induced current thus reducing Ohmic (I^2^R) heating in the neural probe’s conductive traces.

In our previous work [35], we developed a monolithic Michigan style neural probe from 3C-SiC called the “*all*-SiC” INI. The *all*-SiC neural probe was monolithically fabricated from 3C-SiC and amorphous SiC (*a*-SiC) without the use of polymers or metals except on the contact pads that contained gold (Au), which are outside of the brain. The fabrication method eliminates any metallic parts in contact with tissue, thus increasing the tissue compatibility and decreasing the electrical conductivity of the shank part of the probe. In the present work, the magnetic compatibility of 3C-SiC, in comparison with Pt and Si, was studied. Electromagnetic and thermal finite element method (FEM) simulations in ANSYS Multiphysics, and a Fourier based method to calculate the **B_0_** field shift caused by Δχ in MATLAB, were developed. In addition, saline gel phantom experiments under 7 T MRI were utilized to both validate the simulations and experimentally compare the MRI compatibility of the *all*-SiC neural implants to Si and Pt INI materials. Finally, image artifacts surrounding the *all*-SiC probe were observed and simulated.

## 2. Materials and Methods

### 2.1. Sample Microfabrication

The samples fabricated for this study include 3C-SiC and Si samples of different doping type and concentration, and Pt deposited on Si and polymer substrates. These materials are either used commercially in neural implants or in our monolithic SiC neural probes. The samples tested for image artifact visualization were a rectangular shaped electrode (15 × 4 mm) representing stimulating and recording electrodes used in neural probes. Another set of samples were fabricated on similar sized substrates but contained three parallel 100 µm wide single-ended electrodes on the substrate. The length of these electrodes was 4, 6, and 8 mm. This design was chosen to study the effect of electrode length on both image artifacts and resulting SAR. It also helped us determine how EM coupling between the electrodes can influence image artifacts and SAR distribution surrounding each electrode. The SAR and heat predictions using FEM simulations provided a better picture of the EM interactions between the electrodes and **B_1_** field in the MRI. Unfortunately, in this study, the temperature resolution of the available fiber-optic temperature probes (SA Instruments, Inc., Stony Brook, NY, USA) was insufficient to accurately measure volumetric heating in the tissue phantom; thus, simulation of the EM interactions proved very useful in our overall understanding of the interaction.

**Figure 1:**
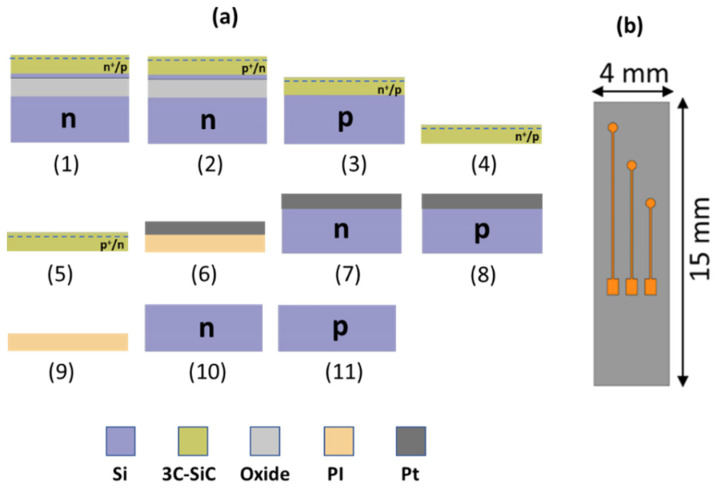
Samples fabricated for the MRI experiments. (**a**) Sample cross section for image artifact analysis. Sample number (1) through (11) correspond to those of Section 2.1. Si (silicon), 3C-SiC (cubic SiC), PI (polyimide), and Pt (platinum) were the materials used as shown. (**b**) Top view of the three electrode test devices (4, 6, and 8 mm long) used for SAR analysis for reference.

The samples fabricated for the MRI experiments are shown in Figure 1 and are as follows:

(1) 3C-SiC epi-film (n^+^/p) on a silicon on insulator (SOI) substrate.

(2) 3C-SiC epi-film (p^+^/n) on a similar silicon on insulator (SOI) substrate.

(3) 3C-SiC epi-film (n^+^/p) on a p-type Si substrate.

(4) Free-standing 3C-SiC (n^+^/p): (1) after oxide and residual Si removal from backside.

(5) Free-standing 3C-SiC (p^+^/n): (2) after oxide and residual Si removal from backside.

(6), (7), (8) Pt film ~ 1 μm thick on PI film and n- and p-type Si substrate, respectively.

(9), (10), (11) PI (Kapton tape), n-type Si, and p-type Si, respectively.

**Figure 2:**
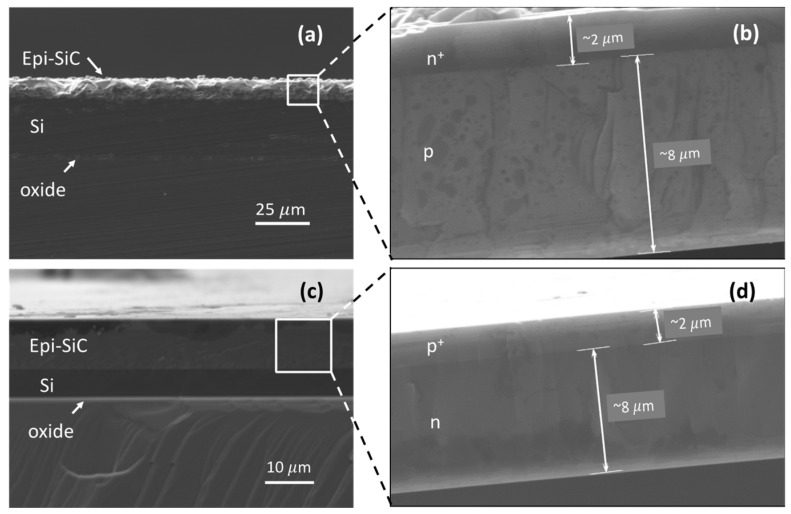
Cross section SEM micrographs of the 3C-SiC samples. (**a**) Cross section view of the n^+^/p 3C-SiC on SOI substrate. (**b**) Free-standing n^+^/p sample after etching oxide in HF and Si in KOH solution. (**c**) Cross section view of the p^+^/n 3C-SiC on SOI substrate. (**d**) Free-standing p^+^/n sample.

Fabrication details of the 3C-SiC film stacks were provided in our previous work [35]. The doping density of the 3C-SiC conductive traces (n^+^ on the n^+^/p wafer and p^+^ on the p^+^/n wafer) was measured to be ~10^19^ dopants/cm^3^. The samples were diced into 15 × 4 mm pieces and used as base reference electrodes. Figure 2 shows cross-section SEM micrographs of the 3C-SiC samples before and after oxide and Si etching from the film stacks.

Pt was deposited on polyimide (PI), n-type, and p-type Si substrates. This combination of substrates was selected to investigate the influence of substrate material on image artifacts surrounding the Pt electrodes. Nevertheless, in the case of neural probes, Pt is typically deposited on a substrate that is either Si or a polymer film such as PI [14].

A Pt film (~1 μm thick) was deposited via E-beam evaporation on two p- and n-type Si substrates. The resistivity of the p- and n-type substrate was ~9 and ~0.5 Ω-cm, respectively. Prior to Pt deposition, a very thin (20 nm) layer of titanium (Ti) was deposited, which acts as a Pt adhesion layer [46]. Another set of samples was fabricated with three Pt electrodes on the substrate. Using a metal lift-off photolithography process, Pt was patterned as illustrated in Figure 1b.

### 2.2. Modeling and Simulation

#### 2.2.1. Magnetic Perturbation Simulations

In this section, details of the Fourier-based simulations used to predict the magnetic perturbation caused by Δχ between the sample and the saline gel phantom, and also the FEM simulations to calculate the SAR, **B_1_** field distortion, and the induced heating, are provided.

A Fourier-based method was used and implemented in MATLAB (R2017b, MathWorks, Natick, MA) to predict image artifacts induced by Δχ. The MATLAB codes were written based on the method reported in [32,47]. For simulation purposes, the following materials were studied: Pt, free-standing 3C-SiC, and Si substrates. The background material in which the samples were placed was a saline-based gel phantom. The magnetic susceptibility of these materials is listed in Table 1. Via these simulations, the **B_0_** field perturbation induced by each material was estimated. The magnetic interaction between a stack of layers was not captured in these simulations, but rather they were experimentally measured later in this work. By combining the simulation and experimental results, the image artifact patterns observed during the MRI experiments from individual material layers, and the interaction between them and the background material, are better understood. The experiments also helped verify and validate the numerical SAR models, a very important point, as direct measurement of a minute temperature rise in the tissue phantom was not possible due to resolution limits of available temperature probes.

In these simulations, the **B_0_** field was set to 7 T, the field of view (FOV) was 25.6 mm × 25.6 mm × 25.6 mm, sample length and width were 15 mm and 4 mm, respectively, and the voxel size was 0.1 mm × 0.1 mm × 0.1 mm. More details about the simulation codes and variables can be found elsewhere [48].

#### 2.2.2. Finite Element Method Simulations

An essential benefit of performing simulations is to control all aspects of a physical phenomenon, which is often not possible during experiments. The goal of these simulations was to compare the SAR distribution surrounding the various materials under RF excitation in the MRI. However, since the length of the samples was short (relative to the wavelength of the RF signal inside the tissue phantom), the electromagnetic coupling consequently was reduced, which, as a result, decreased the induced heating effect. Although low in intensity, the heat is expected to increase dramatically when the length of the samples becomes comparable to the wavelength of the RF signals inside the MRI [49]. In addition, using FEM EM simulations, the **B_1_** field distortion profile surrounding the samples can be estimated and its correlation to measured image artifacts understood.

**Figure 3:**
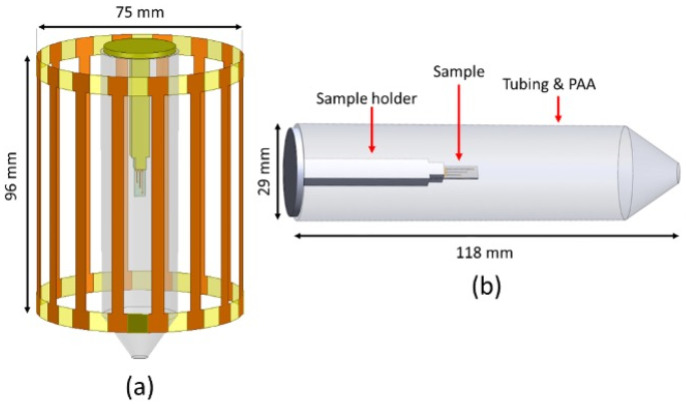
Illustration of ANSYS model for electromagnetic and thermal simulations. (**a**) High-pass birdcage designed for the simulations with 16 rungs tuned to ~300 MHz. (**b**) Sample tube filled with PAA phantom and a 3D-printed PLA sample holder.

ANSYS HFSS (19.2, Ansys, Inc., Canonsburg, PA) was used to perform the FEM electromagnetic simulations. For simulation purposes, a 16-leg high-pass birdcage resonator of 75 mm diameter and 96 mm length was designed and tuned at ~300 MHz in HFSS. An auto-open region radiation boundary condition was chosen to surround the birdcage resonator. Although we are aware that this boundary condition might result in a slight overestimation of the simulated **B_1_** field due to RF shielding within the bore in MRI scanners [50], we believe this will not change the qualitative results presented here. During experiments (discussed later) the Pt and 3C-SiC samples (Figure 1b,c) were placed inside a tube and fixed in-place using a 3D printed polyacrylic acid (PLA) holder. The tube was filled with the saline gel phantom, and this sample holder was modelled in ANSYS HFSS (Figure 3). The EM simulation was set to have a convergence criterion of ΔS<0.01, when solving for Maxwell’s equations [9]. The simulations were run on 24 CPU cores (2x Intel^®^ Xeon^®^ CPU E5-2650 v4 @ 2.20GHz with 5 × 64 GB of RAM) within the USF research computing center.

As mentioned previously, accurate temperature rise measurement around the samples using the commercial temperature probes available to us was not possible. To resolve this issue, we measured the temperature rise at two different locations inside the phantom when the electrode samples were absent, during MRI acquisition as described below in Section 2.3. Comparing these measurements with the EM simulation results, we were able to tune the initial and boundary conditions of the simulation and, ultimately, to verify the simulation results. In order to estimate the temperature rise, the EM simulations were linked to thermal simulations in ANSYS Workbench™ and ANSYS Mechanical™. In ANSYS Mechanical a thermal radiation boundary condition (emissivity of 0.95 [51,52]) was applied to the outer surface of the tube. The **B_1_** field distribution inside the tube was also estimated in these simulations and later used to estimate the SAR distribution surrounding the samples.

### 2.3. MRI Experiments

The MRI experiments were performed in a 7T horizontal magnet (Agilent-Technologies) with Bruker electronics (BioSpec AV3HD) and a 35-mm M2M birdcage coil (M2M imaging corp). The aforementioned samples were inserted into a saline gel (polyacrylic acid, PAA) to mimic a human tissue phantom (Figure 3).

#### 2.3.1. Saline Gel Phantom Preparation

A saline gel was used as the tissue phantom to simulate the electrical, thermal, and magnetic properties of human brain tissue. In order to accurately mimic the properties of human tissue, gel synthesis was precisely followed according to the relevant standards [49]. The phantom was made by mixing polyacrylic acid (PAA, Sigma-Aldrich, St. Louis, MO, USA) and pure NaCl (reagent grade, purity >99%) dissolved in distilled water. The ratio of NaCl to water was 1.32 g/L, while the value for PAA and water was 10 g/L. The electrical conductivity and dielectric constant of the mixture were measured using a network analyzer (Keysight, Santa Rosa, CA, USA) and a dielectric probe. The measured electrical conductivity of the phantom at 25 °C and 300 MHz was σ ≈ 0.5 S/m, while its relative dielectric constant was $\varepsilon_{r}$ ≈ 70. According to the applicable standards [49], this recipe should result in a diffusivity of 1.3 × 10^−7^ m^2^/s and a heat capacity of 4150 J/Kg-C, which are equal to the thermal properties of human brain tissue.

#### 2.3.2. MRI Image Artifact Experiments

**Figure 4:**
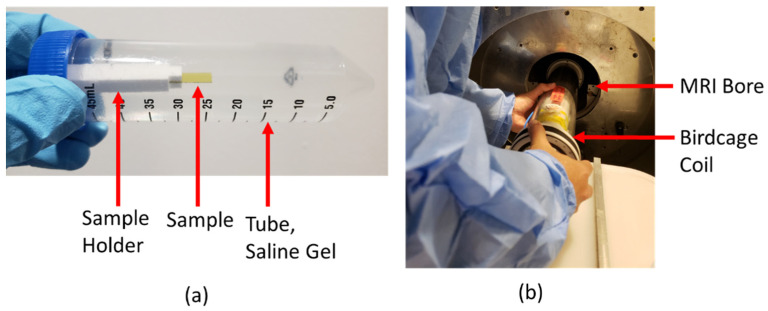
MRI experimental details. (**a**) Plastic tube filled with saline gel phantom, test sample, and 3D-printed polylactic acid (PLA) holder. Holder glued to the blue screw cap and the sample to the PLA holder. (**b**) Coil insertion into the MRI bore used for all MRI experiments. The sample tube was first centered in the coil. Note the absence of air bubbles in the phantom.

The apparatus used for image artifact measurement is shown in Figure 4a,b. The setup consisted of a plastic tube that was filled with the saline gel. A 3D-printed polylactic acid (PLA) holder was used to place the sample inside the plastic tube and was glued to the screw cap of the tube. Prior to insertion into the tube, the sample under test was glued to the PLA holder. The cap was carefully tightened, the gel checked for the absence of air bubbles, and the tube centered inside the RF-coil in the MRI bore.

T2-weighted axial and coronal images of the devices were obtained for image artifact measurement with a RARE (Rapid Acquisition with Relaxation Enhancement) sequence of TR/TE 2220/32 ms, nine slices, and a slice thickness of 0.3 mm. The FOV of the sequence was 35 mm × 35 mm and 256 × 256 pixels. The location of the tubes was carefully inspected to ensure consistent placement in the coil for each measurement.

#### 2.3.3. MRI Induced Heat Experiments

For FEM simulation validation purposes, a similar setup to the one used to measure induced heating was used without the electrode samples. The sample holder was designed to encompass two fiber-optic temperature probes (SA Instruments, Inc., Stony Brook, NY, USA) to measure the temperature rise at two locations inside the tube. One of the fiber-optic probes was at the center of the tube near the sample edge, while the other probe was placed further away from the center, closer to the inner wall of the plastic tube.

A 15 min long MRI scan was performed for heat accumulation with a T1-weighted RARE sequence of TR/TE 440/11 ms, four averages, four repetitions, and 11 slices, with the slice thickness of 0.5 mm. The FOV of the sequence was 30 mm × 30 mm and with 256 × 256 pixels.

## 3. Results

### 3.1. Image Artifacts

Image artifacts can be noticeable in all directions surrounding the sample. They can be either darker or brighter than the background depending on Δχ, which is the difference between the saline gel phantom and the sample magnetic susceptibility. Additionally, other phenomenon may play a role in determining the severity and contrast of image artifacts, such as the interaction between the sample’s conductive parts and the MRI **B_1_** field. This may lead to **B_1_** field distortion surrounding the samples.

**Figure 5:**
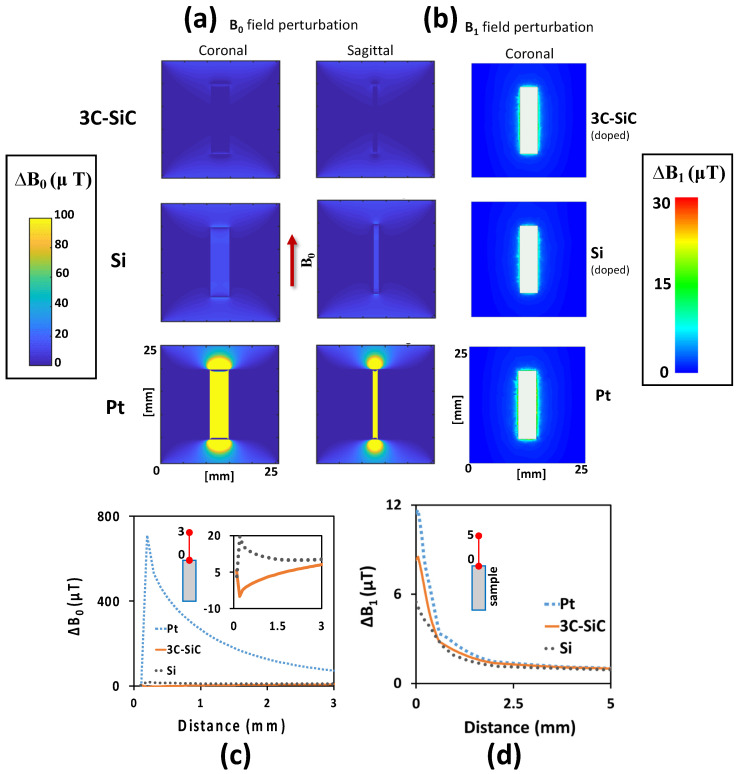
Fourier-based simulations to calculate the **B_0_** field shift caused by the magnetic susceptibility difference (Δχ) between the saline gel phantom and the sample, and EM simulations to estimate the **B_1_** field perturbation surrounding the samples. (**a**) Two coronal and sagittal views of each material are presented. The red arrow shows the direction of the static magnetic field (**B_0_**). The lowest simulated **B_0_** field distortion was observed in 3C-SiC and the highest in Pt. This is due to the negligible Δχ between the saline gel phantom and 3C-SiC material. All simulations are for 7 T MRI. (**b**) **B_1_** field perturbation surrounding the samples estimated using FEM EM simulations for the 3C-SiC (~10^19^ dopants/cm^3^), doped Si (~10^18^ dopants/cm^3^), and Pt samples. (**c**) Δ **B_0_** along the path extending from the sample midpoint of the top edge to 3 mm away from the 3C-SiC, Pt, and Si samples. (**d**) Δ **B_1_** along the same path as (**c**).

Fortunately, the influence of these two phenomena can easily be separated via numerical simulations. The simulations can then be compared to MRI experimental data for a better understanding of the composite interactions involved. Fourier-based simulations estimating **B_0_** field perturbation due to Δχ, and EM simulations estimating **B_1_** field perturbation, are shown in Figure 5. The results indicate a shift in the **B_0_** field, as well as a **B_1_** field perturbation, surrounding the samples. As expected, regarding the value of χ for 3C-SiC compared to Pt and Si, the 3C-SiC sample displayed a lower amount of **B_0_** field shift surrounding the sample while Pt displayed the highest. Figure 5c shows Δ**B_0_** along the line extending from the sample midpoint of the top edge to 3 mm away from the 3C-SiC, Pt, and Si samples. As shown, Δ**B_0_** caused by the 3C-SiC sample is nearly zero, while the Pt and Si samples show higher Δ**B_0_** values along this path. Figure 5d also shows Δ**B_1_** along the same path up to 5 mm away from the sample.

**Figure 6:**
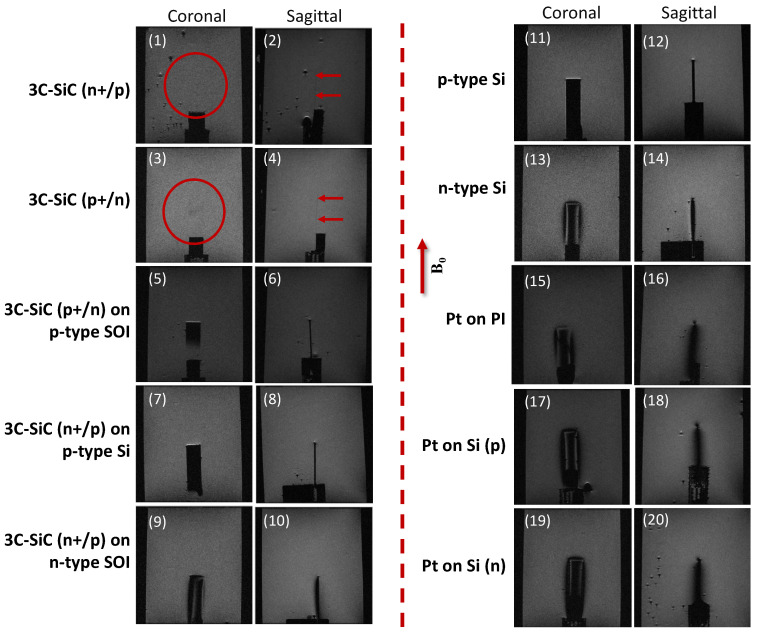
MRI experimental scan images indicating the intensity of the image artifacts in the samples previously simulated. **B_0_** field direction shown by arrow in figure. (1) Coronal view of free-standing 3C-SiC (n+/p). As seen, this thin sample (~10 µm) was almost invisible in the scans. This is also true for the sagittal view of the similar sample (2). (3,4) 3C-SiC sample (n+/p) did not display any footprint in the scans. Image artifacts were minimum for all 3C-SiC samples grown on p-type Si substrates (5–8). However, all of these samples were visible in the MRI scans when the Si substrate remained on the sample. On the other hand, 3C-SiC on n-type Si created image artifacts when the Si substrate remained on the sample (9,10). Opposite to the p-type Si substrate (11,12) that did not show any image artifacts, the n-type Si substrate generated comparatively high image artifacts (13,14). Pt showed severe image artifacts, regardless of the substrate used (15–20). All experiments for 7T MRI.

The results of the MRI experiments are presented in Figure 6. As observed in this figure, all materials tested, except 3C-SiC, showed extended image artifacts surrounding the samples. The free-standing 3C-SiC samples, for both p^+^/n and n^+^/p cases, were nearly invisible in the MRI images, as seen in the figure. Conversely, image artifacts surrounding the Pt samples covered the whole device and distorted its shape. For the silicon substrates the severity of the image artifacts was also correlated with the doping type (n or p). This can be seen for the p-type Si substrate, which did not show any extended image artifacts caused by magnetic field distortion. This will be discussed more in the discussion section.

### 3.2. Induced Heat and SAR

**Figure 7:**
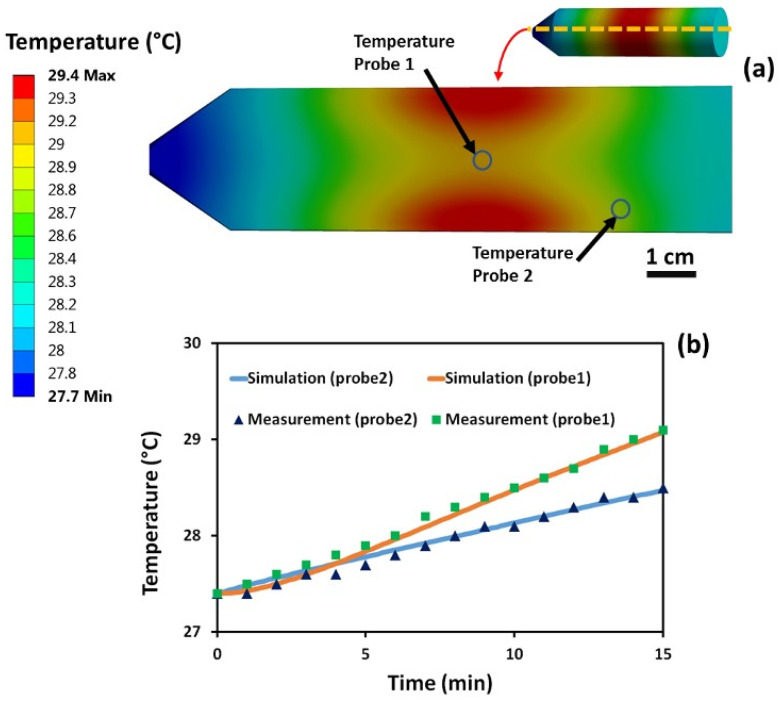
Measured and simulated gel phantom temperature rise without any samples present. (**a**) Cross section view of the sample tube simulated temperature profile after 15 min of exposure to ~300 MHz RF field excitation. (**b**) Measured and simulated temperature profiles at probe 1 (probe tip) and probe 2 (tissue phantom) locations. Initial temperature was 27.4 °C, and after 15 min exposure to MRI scan, it increased to 29.1 °C at the probe 1 location and to 28.5 °C at the probe 2 location. Estimated FEM value at the probe 1 location was 29.08 °C and at the probe 2 location 28.47 °C, which is in a very good agreement with experimental results.

In order to validate the simulations, the temperature inside the saline gel phantom (no sample present) was measured using fiber-optic temperature probes, as explained earlier. These results were then compared with the FEM EM and thermal simulation results for this setup. The measured and estimated results for temperature rise during a 15 min MRI scan are presented in Figure 7.

The initial temperature of the phantom was 27.4 °C and, after 15 min of MRI exposure, the temperature in the middle of the tube where the sample was located (probe 1), increased to 29.1 °C (ΔT = +1.7 °C). The phantom temperature at the probe 2 location after 15 min exposure increased from 27.4 to 28.5 °C. By way of comparison the estimated value from FEM simulation at probe 1 was 29.08 °C, and at probe 2 was 28.47 °C, as shown in Figure 7b. In these simulations, the goal was to keep the final simulated temperatures as close as possible to the measurements when both the simulations and the measurements started from the same temperature. The negligible final temperature differences between the measurements and the simulations was due to the simulations accuracy. Using these simulation results, the intensity and distribution of **B_1_** inside the plastic tube was estimated and used in the following simulations.

**Figure 8:**
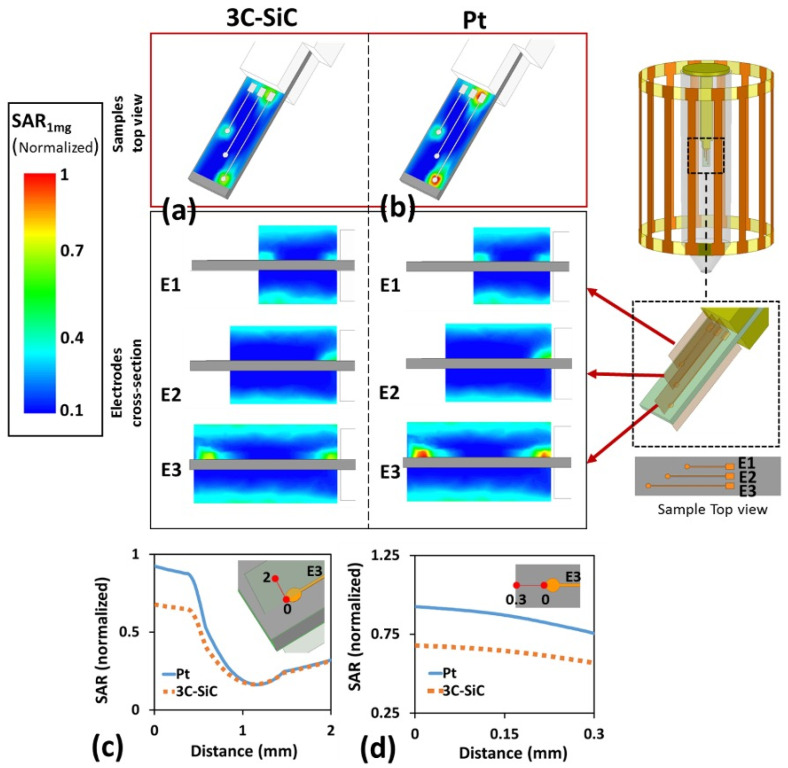
Simulated normalized SAR_1mg_ around the Pt and 3C-SiC (both on Si substrate) samples containing three electrodes. E1, E2, and E3 denote short, medium, and long electrode, respectively. The results are normalized with the maximum SAR_1mg_ value that occurs at the vicinity of the largest Pt electrode. (**a**) Estimated normalized SAR_1mg_ for 3C-SiC via FEM electromagnetic simulations in ANSYS HFSS. Both top (top image) and cross-section (bottom images) views for each electrode illustrated. Maximum estimated SAR_1mg_ for 3C-SiC appeared at the tip of the longest electrode, while the averaged **B_1_** at the coil isocenter was ~10 µT. (**b**) Estimated normalized SAR_1mg_ distribution for the Pt sample. Estimated normalized SAR_1mg_ along the path starting from the tip of the E3 electrode to 2 mm away in the vertical plane (red line) as indicated in plot (**c**) and to 0.3 mm away in the horizontal plane (red line) as shown in plot (**d**). Maximum SAR_1mg_ occurred at tip of longest electrode. Results indicate a ~30% reduction in the maximum SAR_1mg_ for 3C-SiC compared to Pt.

The next set of simulations were performed to estimate the SAR distribution around each electrode for the three electrode samples. The results for the FEM simulations indicating the SAR_1mg_ (specific absorption rate averaged over 1 mg of phantom) distribution in two Pt and 3C-SiC samples are shown in Figure 8.

As shown in Figure 8, the highest SAR_1mg_ value occurred at the sample extremities where the electric current flows out of the electrodes. However, the hot spot area in the 3C-SiC sample showed considerably less intensity compared to the Pt sample. While the estimated SAR_1mg_ was very low in both cases, if the size of the electrodes is scaled up the SAR_1mg_, values will also increase. The SAR_1mg_ value for 3C-SiC showed an ~30% reduction compared to Pt.

### 3.3. C-SiC Neural Probe under 7 T MRI

**Figure 9:**
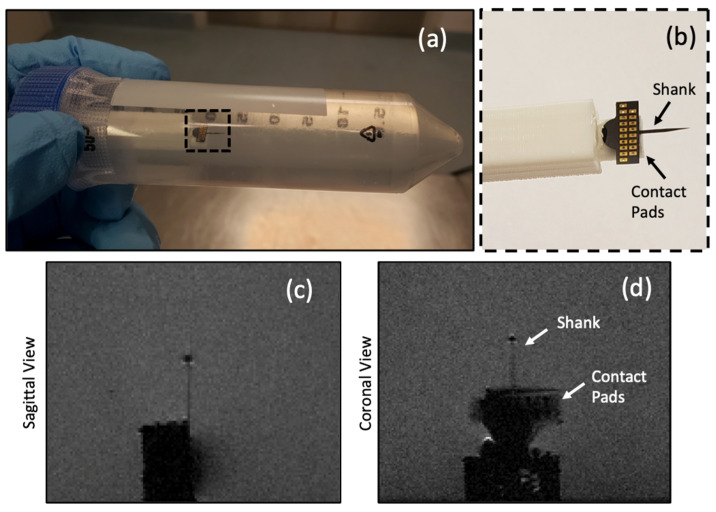
Experimental details for the fabricated *all*-SiC probe under 7 T MRI scanning. The same MRI sequence for image artifact analysis was used. (**a**) Photograph of the experimental setup consisting of the PLA holder, the plastic tube, the saline gel phantom, and a functional *all*-SiC neural probe. (**b**) A close-up image of the *all*-SiC neural probe glued on the PLA holder. (**c**) Sagittal view of the probe showing a slight circular image artifact. (**d**) Coronal view of the probe. The shank generated less image artifacts than the metallic pads, as expected, which would be outside of the brain during probe use.

For this experiment, the fabricated *all*-SiC probe was glued to the PLA holder and placed inside the plastic tube along with the saline gel phantom. The same MRI sequence used for measuring the image artifacts was then employed. The setup along with the sagittal and coronal images of the probe are shown in Figure 9. The shank of the *all*-SiC probe showed little artifact generation, while the metal pads (Ti/Pt) generated more image artifacts, as expected. This is not an issue in an actual implant, as the pad area is outside of the body and has minimal to no contact with intracranial regions. It is noteworthy that the residual n-type Si layer was not etched from the backside of the fabricated *all*-SiC probe for this experiment. Therefore, some level of artifacts were expected due to this residual Si (~27 µm thick) film.

## 4. Discussion

In the present work, the MRI compatibility of single crystalline cubic silicon carbide (3C-SiC), a novel material used for implantable neural probes, was studied. It has been hypothesized that a brain implant fabricated monolithically from SiC may remarkably improve the MRI compatibility of implants and allow for its use at ultra-high fields. In order to evaluate the MRI compatibility of *all*-SiC neural implants in terms of induced heating and image artifacts, numerical simulations and a 7T MRI were used in this work. Silicon, of various doping types and concentrations, and platinum were used as reference materials to compare the MRI compatibility of *all*-SiC implants to. The MRI compatibility assessment in this work was limited to the intensity of image artifacts surrounding the samples, as well as the induced heating and SAR distribution under RF excitation in the MRI. Various samples were fabricated from SiC, Si, Pt, and Polyimide (PI). Si and PI were mainly used as the substrate to deposit Pt on, while Si and silicon on insulator (SOI) were used to grow thin epitaxial films of 3C-SiC on.

The current work was designed to compare the MRI induced heating and image artifacts caused by the material properties used in intracranial implants. However, it should be noted that in many deep brain implants, the implant lead is the main safety concern [8]. Fortunately, the safety issue with the lead may be minimized in wireless microelectrode arrays, when extracranial and intracranial leads are eliminated. In addition, it is feasible to use surface RF coils at the surface of the brain instead of large, volumetric RF coils to perform the MRI. In this case, the RF field interactions with the rest of the device may be reduced. In the future work, in order to better understand the compliance in a human MRI tool, we will test the MRI compatibility of the entire device.

The measured and simulated results showed adequate agreement to support our hypothesis that 3C-SiC based neural probes may indeed be compatible with 7 T MRI and thus worthy of further technical development.

To support the developed simulations the birdcage coil was simulated in HFSS. The measured **B_1_** field distribution and temperature rise profiles were logically similar to the ones obtained experimentally, thus allowing for a reasonable comparison between the simulated and experimental data. Thermal simulations utilized energy loss from the EM simulations as the input and, by incorporating suitable thermal boundary and initial conditions, estimated the temperature rise profile during 15 min MRI scans. The estimated **B_1_** field map measured with this process was used to estimate the SAR distribution surrounding various samples.

In order to estimate the image artifacts caused by any magnetic susceptibility difference (∆χ) between the samples and the tissue phantom, single layer substrate samples were used in the simulations to compare with experiments in which a combination of materials were tested. This approach narrowed down the possible source of observed image artifacts induced by a particular material, since the image artifacts were generated from a single material. Combining the simulated and the experimental results, one can have a better understanding of the role of each material in the formation of any image artifacts in each fabricated sample. Then it was possible to infer how a stack of such materials will behave when they are integrated into a single device.

One of the observations during this work was the difference in magnetic field perturbation for p- and n-type silicon. The experiments in the current work indicated that the magnetic susceptibility of silicon, and consequently the extent to which it causes magnetic field perturbation and image artifacts under MRI, is highly dependent on doping type. As observed in the experimental results shown in Figure 6, p-type Si induced less image artifacts, while n-type Si displayed severe image artifacts under MRI. The magnetic susceptibility of Si, and its dependence on doping type and concentration, is well documented [43,44,53]. However, a dependence on doping concentration and type was not observed in epitaxially grown 3C-SiC films used in these experiments. The very thin nature of the films, and its magnetic susceptibility being very similar to brain tissue caused negligible image artifacts, as reported elsewhere [32]. Due to the high mechanical strength, flexibility, and chemical stability of 3C-SiC, it was possible to fabricate the samples, through a monolithic fabrication method, into very thin (~10 µm) devices.

The induced heat in the current work was estimated using Multiphysics FEM simulations, and some experimental measurements were used to validate the simulation results. Induced sample heating, specifically in the 3C-SiC samples, was expected to be lower than the resolution of the available fiberoptic temperature probes. Therefore, the measurements focused solely on temperature rise differences between the samples, since absolute measurement was not possible. At the same time, validation of the simulations needed to be performed to ensure the validity of the simulated data. For this reason, the focus of temperature measurement during MRI analysis was, in fact, restricted to measuring the temperature rise inside the phantom at certain locations and comparing it with the corresponding numerical simulations.

As seen in Figure 8, the simulated results indicated a meaningful difference in the SAR distribution between the Pt and 3C-SiC samples. The simulations predicted a ~30% lower maximum SAR_1mg_ for 3C-SiC comparing to Pt. Although both samples experienced very low amounts of SAR, in the case of a longer electrode, it is believed that this difference would be more evident.

Another important point observed during the MRI experiments was the very low level of image artifacts measured for the fabricated *all*-SiC neural probe. The probe showed a very small footprint during MRI scanning. However, the tip of the probe had visible dark image artifacts. The effect of the shape and sharp points in creating image artifacts has been studied elsewhere [32], where it is believed that sharp points can increase image artifacts under MRI. However, one of the reasons that the probe showed some visibility during MRI imaging was believed to be due to the thin (~27 µm) Si substrate film that remained on the probe backside to support the shank during probe handling (for comparison the 3C-SiC layers were ~10 µm in total thickness). Prior to probe implantation, this thin Si layer will be etched away, and we believe the resulting image artifacts will be considerably reduced, and this will be confirmed in follow-on research.

In order to evaluate MRI compliance of the fabricated *all*-SiC neural probes, the probes will ultimately be implanted inside rodent intracranial regions. Negligible image artifacts in a brain are expected in-vivo as predicted by the phantom experiments. Nevertheless, this must be tested, and post-MRI tissue histology performed to assess any tissue damage resulting from MRI scans with respect to a control implant. In the case of the safety of 3C-SiC invasive intracranial implants in human patients, more phantom experiments need to be designed and performed that not only address safety concerns regarding MRI static field strength and SAR limits, but also the MRI gradient fields. In these future experiments, all the implant parts, including the leads, must be tested inside a human MRI bore and RF coil. Only after these experiments are conducted will the MRI compliance of *all*-SiC probes constructed of 3C-SiC under 7 T MRI fields be known.

One of the main applications for an MRI compatible neural implant is deep brain stimulation (DBS). DBS electrodes are larger than intracortical electrodes, and consequently are more prone to MRI induced heating and tissue damage. As MRI is broadly used in pre- and post-surgery DBS placement, developing an MRI compatible DBS lead is very important. 3C-SiC appears to be an excellent candidate for developing an MRI compatible DBS lead, as a monolithic 3C-SiC implant not only showed a high compatibility in this work, but its mechanical and chemical stability, and biocompatibility, may increase the lifetime of DBS implants [54]. The MRI compatibility of DBS leads can be evaluated using the numerical simulations and MRI experiments introduced in this work.

Based on the compelling data presented from this work, further technical development of an *all*-SiC INI appears to be warranted, as well as the development of an *all*-SiC DBS lead. Our hypothesis is that an *all*-SiC neural implant may prove to be compatible with ultra-high field MRI diagnostics, and follow-on in-vivo studies are planned to ascertain the validity of this hypothesis.

## Figures and Tables

**Table 1:** Properties of materials used to calculate MRI induced image artifacts and heating.

Material	Electric Conductivity (×10^6^ S/m)	Thermal Conductivity (W/m-K)	Magnetic Susceptibility χ_v_ (ppm)
Human Tissue	0.5 × 10^−6^	0.4	–9.05
Doped SiC (~10^19^ dopants/cm^3^)	~0.17	360	–12.87
Doped Si (~10^18^ dopants/cm^3^)	~0.1	148	–4.2
Platinum	9.3	71	279
